# Medical Devices in Obesity Treatment

**DOI:** 10.1007/s11892-019-1217-3

**Published:** 2019-08-30

**Authors:** Aruchuna Ruban, Akash Doshi, Erika Lam, Julian P. Teare

**Affiliations:** 1 Imperial College London, St Mary’s Campus, London, W2 1NY UK; 2 Department of Surgery & Cancer, Homerton University Hospital, London, E9 6SR UK

**Keywords:** Obesity, Bariatric surgery, Weight loss, Body mass index, Endoscopy, Gastric, Duodenal

## Abstract

**Purpose of Review:**

Obesity is increasing at an alarming rate and now poses a global threat to humankind. In recent years, we have seen the emergence of medical devices to combat the obesity epidemic. These therapeutic strategies are discussed in this review dividing them into gastric and duodenal therapies.

**Recent Findings:**

Traditionally, medical devices for obesity such as the intragastric balloon have focused on reducing gastric size, but more recently there has been a shift towards developing devices that modulate neural and hormonal responses to induce early satiety thus reducing oral intake.

**Summary:**

Medical devices for obesity treatment may have a role in those patients who are struggling to control their weight despite significant lifestyle modifications such as diet and exercise and who decline or are unfit for bariatric surgery. For the wider adoption and integration of these devices in the obesity treatment paradigm, more long-term efficacy and safety data from randomised controlled trials are required.

## Introduction

Obesity is a consequence of a gross imbalance in calories consumed versus calories expended and its prevalence has risen dramatically, almost tripling from 1975 to 2016. [1] Obesity is defined as a body mass index (BMI) of > 30 kg/m^2^ and is associated with the development of co-morbidities including type 2 diabetes, hypertension and sleep apnea [2, 3]. It is estimated globally that 2.8 million people are dying each year as a result of being overweight or obese. Although increasing obesity prevalence is believed to be largely driven by multiple environmental factors including increased consumption of high-calorie foods and a reduction in physical activity, there is wealth of evidence which also points to the genetic susceptibility of developing obesity. [4, 5]

The current paradigm in obesity treatment has traditionally relied upon lifestyle modifications (diet and exercise) and pharmacological treatments which often lead to modest results in terms of weight loss [6, 7]. Low-calorie diets and high-intensity exercise regimes may prove difficult to adhere to and the maintenance of weight loss requires long-term behavioural modifications which are often hard to implement and maintain. [8] There is also a sparsity of pharmacological agents licenced for obesity and these can be associated with intolerable side effects thus affecting patient adherence [9]. Bariatric surgery has become an increasingly popular treatment choice in the management of obesity and is recommended by the International Federation for the Surgery of Obesity and Metabolic Disorders (IFSO) in patients who have not succeeded with medical management [10]. Bariatric surgery results in superior outcomes in terms of weight loss and weight-associated comorbidities compared with non-surgical interventions [11•]. However, bariatric surgery is invasive and irreversible, so it may not be a suitable option for every patient, particularly in those who are frail with multiple co-morbidities or who decline surgery due to the potential risks involved. With demand for surgery remaining high, the investment of more resources is still required not only to meet this demand but also to develop alternative strategies geared at inducing weight loss.

A new trend in obesity management has seen the development of both endoscopic and radiological bariatric devices or techniques which have the added benefit of being less invasive, relatively easier to perform and potentially reversible. This review discusses the current landscape of medical devices for the treatment of obesity and is divided into gastric and duodenal therapies which are summarised in Table 1.

**Table 1:** Summary of medical devices for obesity

Device	Configuration	Proposed mechanism of action	Weight loss outcomes
Gastric therapies			
Intragastric balloon	Endoscopically placed fluid- or air-filled balloon	Restrictive, space-occupying effects causing early satiety and subsequent reduced food intake	14% EWL at 6 months [12]
Aspire Assist	Endoscopically placed silicone percutaneous gastrostomy tube	Aspirating gastric contents reduce the volume of food being transferred to the small intestine subsequently leading to weight loss	12% TBWL at 12 months [13]
Endoscopic sleeve gastroplasty (ESG): Overstitch	Endoscopic suturing system creating a gastric pouch or sleeve	Reducing gastric capacity	15% TBWL at 1 year, 15% at 18 months [14]
Gastric artery embolization	Endovascular injection of microparticles to occlude the left gastric artery	Reduce appetite stimulation and modulate metabolism by suppressing ghrelin	11.5% EWL at 12 months [15]
Vagal nerve blockade	Laparoscopic deployment electrodes at the gastro-oesophageal junction connected to a subcutaneous neuroregulator	Block conduction of the vagus nerve thereby increasing satiety	17–24% EWL at 12 months [16, 17]
Duodenal therapies			
Duodenal jejunal bypass (EndoBarrier)	60-cm duodenal-jejunal sleeve inserted endoscopically into the duodenum	Bile flow modulation and altered flow of nutrients in the small intestine culminating in changes in enteric gut hormones	13% EWL at 6 months [18]
Incisionless magnetic anastomosis system	2 self-forming magnets which join together to form a compression anastomosis between 2 regions of small bowel	Diversion of nutrients to distal small bowel stimulates anorexigenic hormones such as GLP to increase satiety and decrease intake	14% TBWL at 12 months [19]

## Gastric Therapies

### Intragastric Balloon

The intragastric balloon (IGB) is a device that aids weight loss primarily by occupying space, resulting in early satiety and overall reduced food intake. It has been used in the management of obesity since 1985 and thus has excellent data-measuring efficacy and safety profiles [20].

Multiple balloons are in use with FDA or European approval, with materials ranging from silicone polymers to gelatin capsules. The deflated balloons are deployed endoscopically under conscious sedation, then depending on the device filled up to 500 ml of saline or occasionally air via a catheter and remain in situ for 6 months [21]. Endoscopic removal of devices is relatively simple, involving deflation of the balloon followed by retrieval using a grabber or snare. The most common adverse events affecting patients are abdominal pain, nausea and vomiting. Serious adverse events are upper gastrointestinal bleed (2%), balloon migration (1.4%), gastric necrosis (0.3%), perforation (0.1%) and small bowel obstruction (0.08%) [22•].

Indications for an IGB are for weight loss in patients with a BMI > 35 kg/m^2^ who have failed lifestyle measures, pharmacotherapy or for whom bariatric surgery is contraindicated. IGB may also be used to aid weight loss prior to bariatric surgery to reduce intraoperative risk [23]. Given the ease of deployment and simplicity, IGB has relatively few contraindications. These include structural contraindications, such as gastric inflammation/ulceration or a large hiatus hernia > 5 cm or endoscopic risks such as severe cardiorespiratory comorbidities or coagulopathies.

The majority of weight loss occurs within the first 3 months and mean percentage excess weight loss (EWL) at 6 months is 14% but almost half of patients may return to their initial weight at 12 months after removal of the device [12, 24]. For a large proportion of patients, the weight loss is not sustained making it a less favourable intervention as a long-term solution for weight loss.

### Aspiration Therapy

**Fig. 1:**
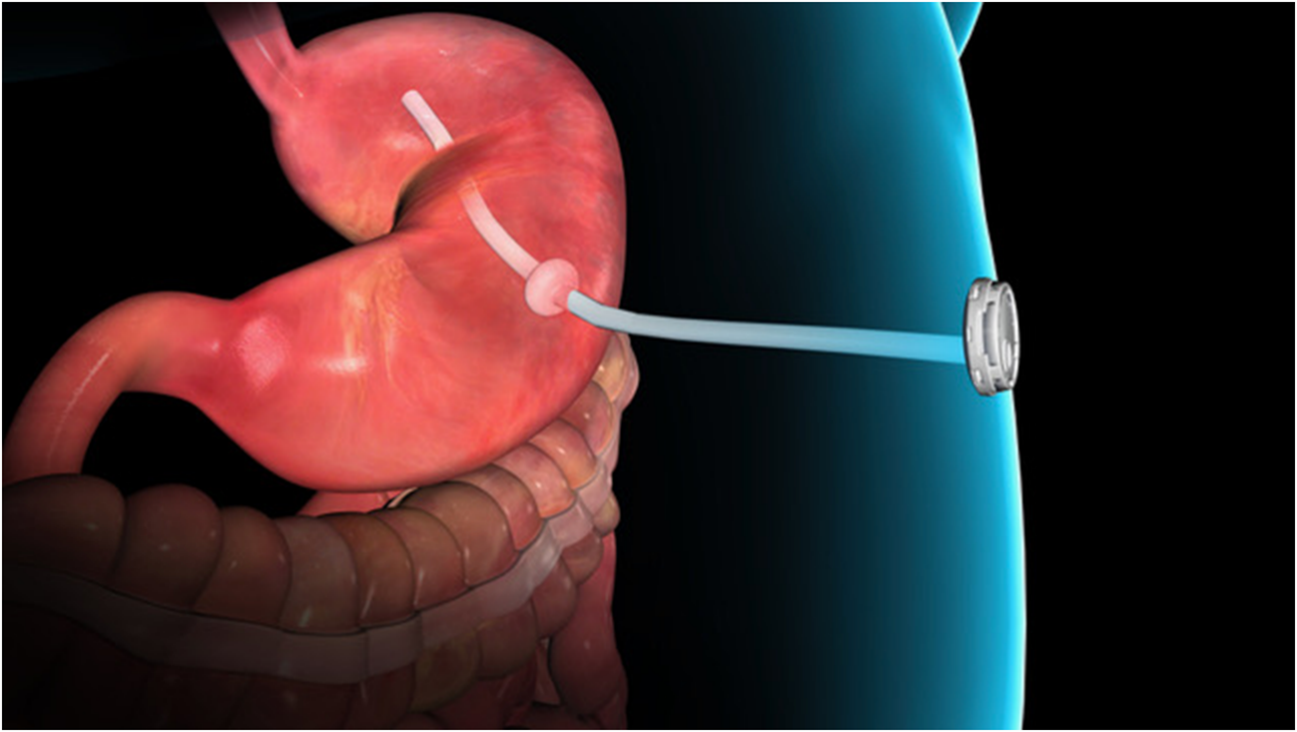
AspireAssist® device. (With permission from Aspire Bariatrics, Inc.)

Aspiration therapy (AT) uses the AspireAssist® device (Fig. 1) which consists of a large-bore percutaneous gastrostomy tube, skin port and attachable drainage system with remote control. The gastrostomy tube is inserted endoscopically under sedation similarly to a percutaneous endoscopic gastrostomy but acts in reverse allowing removal of approximately 30% of gastric content post-meal when the drainage system is attached to the gastrostomy tube via a skin port. Aspiration is performed 20 min after a meal, through cycles of irrigation of water into the stomach, relying on positive pressure from within the stomach to void liquid containing food particles out through the gastrostomy tube [25].

The largest randomised controlled trial of this device involved 207 participants, of which 137 participants received the AspireAssist® in addition to lifestyle counselling, compared with 70 patients in the control group receiving lifestyle counselling only. Using a modified intention to treat analysis at 1 year of follow-up, the TBWL in the AspireAssist® group was 12% compared with 3.5% in the control group respectively [13].

AT is a highly effective and durable method of weight loss that is simple to install and remove. Unlike bariatric surgery, there are few comorbidities that limit candidates for device insertion, although its efficacy depends on user adherence to regular measures such as thorough chewing of food before ingestion. Contraindications to its use include previous abdominal surgery that may significantly increase the risk of implantation of the device and any physical or mental disability (including bulimia or diagnosed binge eating disorder) that might interfere with adherence to therapy. It lends itself well to regular monitoring by health professionals (initially monthly and then three monthly) as users require regular device maintenance such as a tube replacement, providing opportunity to identify problematic eating behaviours that could theoretically be exacerbated by this technique. Other elements of the follow-up visits include monitoring for any electrolyte abnormalities, (hypokalaemia and hyponatraemia) stoma site health as well as lifestyle counselling. A unique advantage of this device is that it can remain in situ for long-term use as evidenced by one observational study which demonstrated a mean EWL 47.9% at 4 years in 12 participants that persisted with AT [26].

### Endoscopic Sleeve Gastroplasty: Overstitch

Endoscopic sleeve gastroplasty utilises an Overstitch device (designed by Apollo Surgery) which is attached to the end of an endoscope to facilitate full thickness continuous suturing to occur [27]. A series of sutures can be deployed from the bottom of the stomach up to the gastro-oesphageal junction which takes about 45 min creating a gastric pouch or sleeve leading to a reduction in gastric size [28]. This mimics the anatomical changes of laparoscopic sleeve gastrectomy (LSG) which is the most commonly performed bariatric surgery. In a prospective observational study of 1000 patients who underwent ESG, the mean percentage total weight loss (TBWL) was 15% at 18 months with only 14% of patients lost to follow-up in this study [14]. These results suggest good durability of the device with promising long-term outcomes in terms of weight loss. However, some patients may need to undergo repeat procedures 2–3 years after the initial procedure to tighten the gastric pouch if weight gain occurs. ESG has some obvious advantages over LSG with the potential to be reversed if necessary and has a lower incidence of gastro-oesophageal reflux disease post-procedure [29]. However, it appears less effective at promoting weight loss than LSG achieving a TBWL of 17% at 6 months compared with 23% with LSG.

### Gastric Artery Embolization

Gastric artery embolization (GAE) is an endovascular technique using a femoral or radial approach to inject 300–500-μm embolic microparticles (Embosphere, Merit Medical Systems, Jordan, UT, USA – in BEAT) to occlude the left gastric artery (LGA). The principle of this technique is to reduce appetite stimulation and modulate metabolism through the suppression of the hunger hormone ghrelin. Ghrelin is a hormone produced mainly in the gastric fundus that is a potent central appetite stimulant and drives positive energy balance [30]. Ghrelin resistance is a key feature of obesity and its production is increased after diet-induced weight loss and reduced after bariatric surgery such as gastric bypass [31]. The LGA is the main supply to the gastric fundus where the majority of ghrelin-producing cells are located.

LGA embolization was first shown to suppress ghrelin secretion in a porcine model [32]. The first human study was a retrospective analysis of weight loss in patients receiving coeliac trunk embolization for UGI bleeding [33]. In 19 participants who underwent LGA embolization, greater weight loss was experienced in the first 3 months compared with controls who underwent embolization of other branches of the coeliac trunk.

The largest single prospective study involved 20 morbidly obese patients who had an EWL of 11.5% at 12 months [15]. Hunger scores were recorded and mean values decreased from baseline throughout follow-up, correlating inversely with weight loss. Of note, ghrelin was not measured amongst the metabolic endpoints in this study. GAE was a well-tolerated procedure, with only minor side effects reported such as epigastric pain, nausea and vomiting that resolved within 48 h of the procedure. At the 2-week follow-up endoscopy, asymptomatic gastric irritation was identified but all had resolved by the 3-month endoscopy.

Initial data for GAE support it as a promising technique to supplement weight loss goals and the reduction of ghrelin [34]. It has a high level of technical feasibility but requires angiographic identification of relevant vasculature supplying the gastric fundus in case of anomalous anatomy and is therefore not feasible in an absent or accessory LGA.

### Vagal Nerve Blockade

Neuromodulation of the vagus nerve as a therapeutic intervention has been in clinical use since 1988 for the treatment of epilepsy and has since been approved to also treat depression providing a long-term safety profile [35]. With respect to obesity, the vagus nerve is thought to play a key role in satiety and autonomic control of the gastrointestinal tract [36]. Vagal nerve blockade (vBloc) is achieved using a pacemaker-like device developed by EnteroMedics. Electrodes are applied to the gastro-oesophageal junction through minimally invasive laparoscopic surgery to block conduction and thus promote and prolong satiety. These connect to a subcutaneously implanted neuroregulator that can be charged transcutaneously.

Following the success of two small-scale studies, two larger scale double-blind randomised studies have been conducted including the ReCharge study which compared vBloc with a sham device [16, 17, 37, 38]. Both groups were also enrolled in regular weight management programmes providing education only with a healthy lifestyle. In 238 participants at 12 months, EWL was 24.4% in the vBloc group vs 15.9% in the sham group. EWL was sustained in the vBloc group at 18 months with 23.5% EWL compared with 10.2% EWL in the sham group [39]. At 24 months, EWL was 21% in the vBloc group [40].

Although minimally invasive surgery is used, VBLOC carries the risk and contraindications of a general anaesthetic at implantation. Common adverse effects associated with the device include abdominal pain, dyspepsia and belching although these resolve in 79% of patients at 18 months [39]. Serious adverse effects are rare and include neuroregulator malfunction or severe pain and vomiting. One patient suffered gastric perforation at explant following withdrawal from the study but all patients made a full recovery with no sequelae.

### Duodenal Therapies

#### Duodenal-Jejunal Bypass Sleeve Liner

The duodenal-jejunal bypass sleeve liner is an endoscopic implant (EndoBarrier) that consists of a 60-cm fluoropolymer impermeable sleeve with nitinol anchors on its proximal end. Designed to mimic a gastric bypass, once deployed endoscopically, the device is anchored in the duodenal bulb and spans the length of the proximal small intestine thus preventing nutrients from being absorbed here. This results in a variety of effects which induce satiety and weight loss including nutrients being delivered distally as well as a modulation of bile flow and gut hormones [41].

The first clinical trials of the EndoBarrier were published in 2008, and since then there have been numerous publications investigating the efficacy, safety and mechanisms of actions of the device in both animal and human trials [42, 43]. Rohde et al. published a systematic review and meta-analyses in 2015 of the EndoBarrier included were 5 RCTs and 10 observational studies [18]. Although the risk of bias was evaluated as high in all studies as none of these RCTs were blinded studies, meta-analysis showed the EndoBarrier had a statistically significant impact on weight loss. Mean differences in body weight and EWL were 5.1 kg and 12.6% respectively compared with dietary intervention.

The EndoBarrier is minimally invasive and is straightforward to implant and remove endoscopically but it is however associated with a number of adverse events such as bleeding, migration and hepatic abscess. In nearly all these cases, patients have recovered without any permanent sequelae and no fatalities have been reported as a direct consequence of EB treatment. Another limitation of the device is that the manufacturer advises a maximum duration of therapy of 1 year so it is unlikely to provide a permanent solution for weight loss therapy.

#### Dual-Path Enteral Bypass: Incisionless Anastomosis System

Most nutrient transport occurs across the villi in the small intestine [44]. In anastomotic enteral bypass, a connection is made between areas of the small intestine to reduce the length available for nutrient absorption and thus aid weight loss. IAS is a new technique using magnets to achieve an anastomosis. Simultaneous enteroscopy and colonoscopy are used to deploy and align self-forming magnets such that they compress the jejunum and ileum together [45, 46]. The procedure takes 1.5–2 h, forming a compression anastomosis in a week, without laparoscopic surgery or sutures and the risks associated with these. Once formed, the magnets painlessly exit the GI tract at a mean duration of 23 days. The first human study included 10 patients with 14.6% total body TBWL at 12 months with no bleeding, leak or other serious complications reported [19]. Long-term data and safety profile from RCTs is awaited.

## Conclusions

The ultimate goal in the development of medical devices for obesity is to create an intervention which is semi-permanent or permanent but associated with little or no side effects. These procedures need to be safe and easy to perform with the efficacy of the device maintained for long treatment periods in order to provide a long-term, viable solution for the treatment of obese patients. Most of the therapies described above are still in their investigational stages and at present are unlikely to displace bariatric surgery in the treatment algorithm of obesity. They do however provide a potential therapeutic strategy in those patients who may be struggling to control their weight despite significant lifestyle modifications such as diet and exercise, but who decline surgery because of the risks associated. These devices may also be used as an adjunct to surgery, to promote initial weight loss in patients prior to them undergoing elective surgery in order to reduce their intraoperative risk. Over the next few years, we expect to see more long-term efficacy and safety data published from randomised controlled trials which may in turn lead to some of these interventions being adopted and integrated into the treatment paradigm of obesity.
